# Supplementary material for: A single, improbable B cell receptor mutation confers potent neutralization against cytomegalovirus
Source: PLoS Pathog. 2023 Jan 20;19(1):e1011107. doi: 10.1371/journal.ppat.1011107 (PMC9891502; doi:10.1371/journal.ppat.1011107)
Supplement: S1 Fig — (PDF) [file ppat.1011107.s001.pdf]

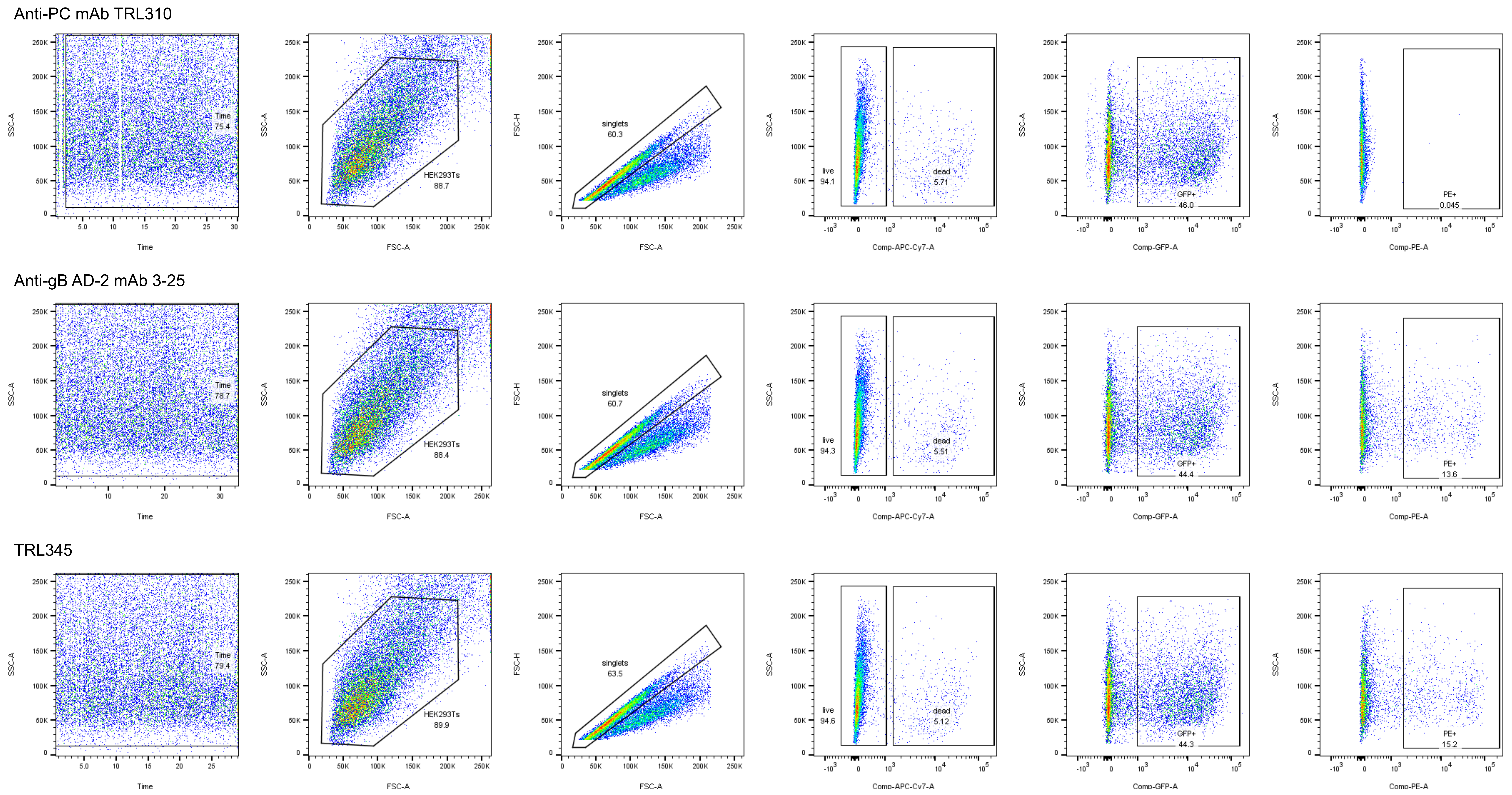

Fig. S1. Gating strategy to determine the % of binding to cell-associated gB. We co-transfected DNA plasmids that separately expressed full-length gB (Towne strain) or GFP. We coincubated gB and GFP-expressing cells with mAbs in a three-point, 10-fold serial mAb dilution, then detected anti-gB AD-2S1 mAb binding with PE-conjugated anti-human IgG Fc. We defined the minimum threshold of positive expression as binding by an anti-CMV pentameric complex mAb "TRL310" and reported the % of GFP-expressing, live HEK293T singlets as "% gB-transfected cell binding."
