# Supplementary material for: A single, improbable B cell receptor mutation confers potent neutralization against cytomegalovirus
Source: PLoS Pathog. 2023 Jan 20;19(1):e1011107. doi: 10.1371/journal.ppat.1011107 (PMC9891502; doi:10.1371/journal.ppat.1011107)
Supplement: S2 Fig — (PDF) [file ppat.1011107.s002.pdf]

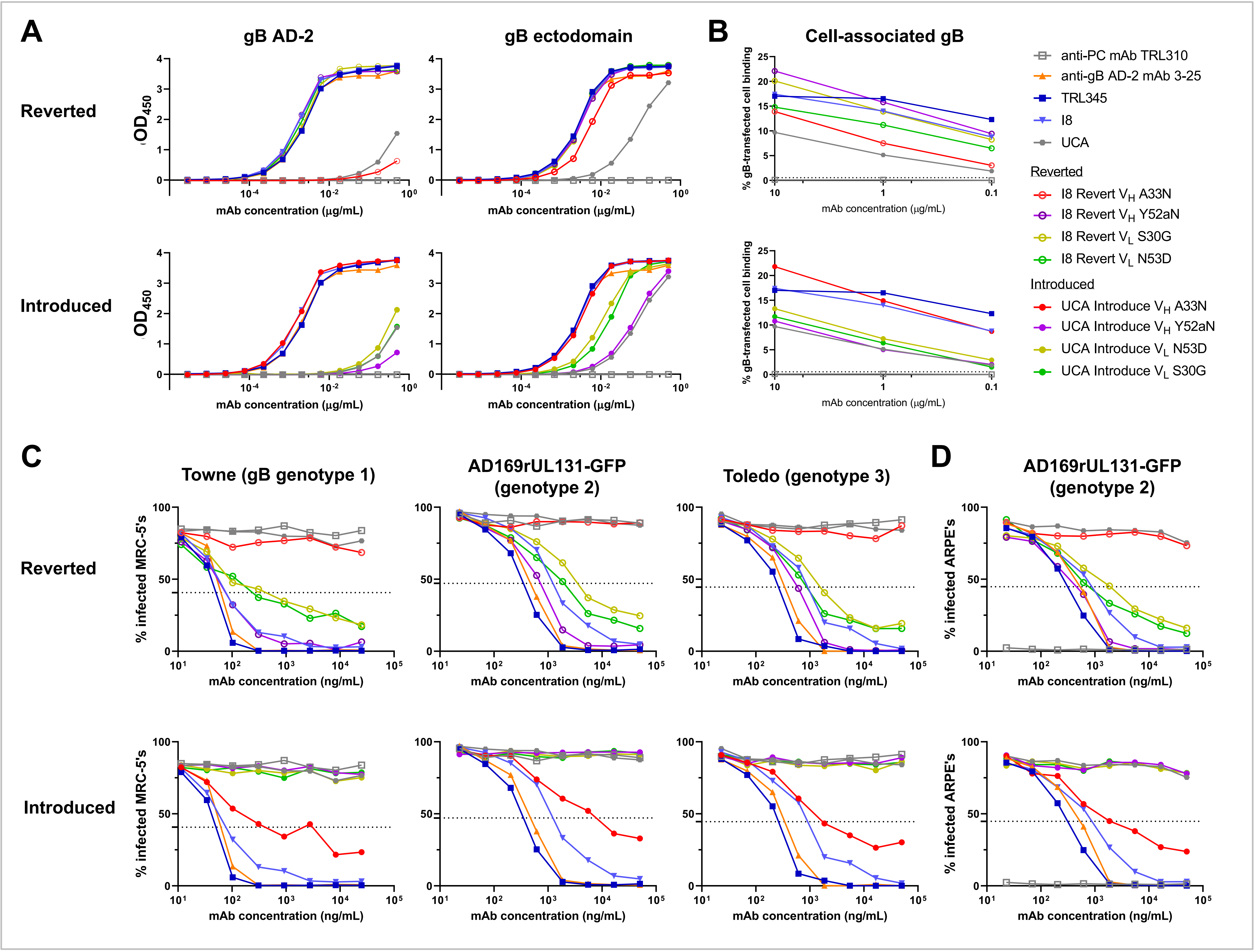

Fig. S2. The VH A33N mutation was both necessary and sufficient for high gB AD-2 binding, binding to cell-associated gB, and neutralization function. We produced the 17 clonally related mAbs of the TRL345 lineage and measured the following:

(A) Binding magnitude to gB AD-21 peptide and gB ectodomain by ELISA.

(B) Binding to cell-associated gB. Binding of mAbs was determined by coincubating mAbs in a serial dilution with HEK293T epithelial cells-transfected with full-length gB and GFP. The % binding was calculated as the % of GFP-expressing cells bound by the anti-gB AD-2 mAb, detected by flow cytometry.

(C) Neutralization function against CMV strains Towne, AD169rUL131-GFP (AD169r), and Toledo on MRC-5 fibroblasts.

(D) Neutralization function against CMV strain AD169rUL131-GFP on ARPE epithelial cells.
