# Supplementary material for: A single, improbable B cell receptor mutation confers potent neutralization against cytomegalovirus
Source: PLoS Pathog. 2023 Jan 20;19(1):e1011107. doi: 10.1371/journal.ppat.1011107 (PMC9891502; doi:10.1371/journal.ppat.1011107)
Supplement: S3 Fig — (PDF) [file ppat.1011107.s003.pdf]

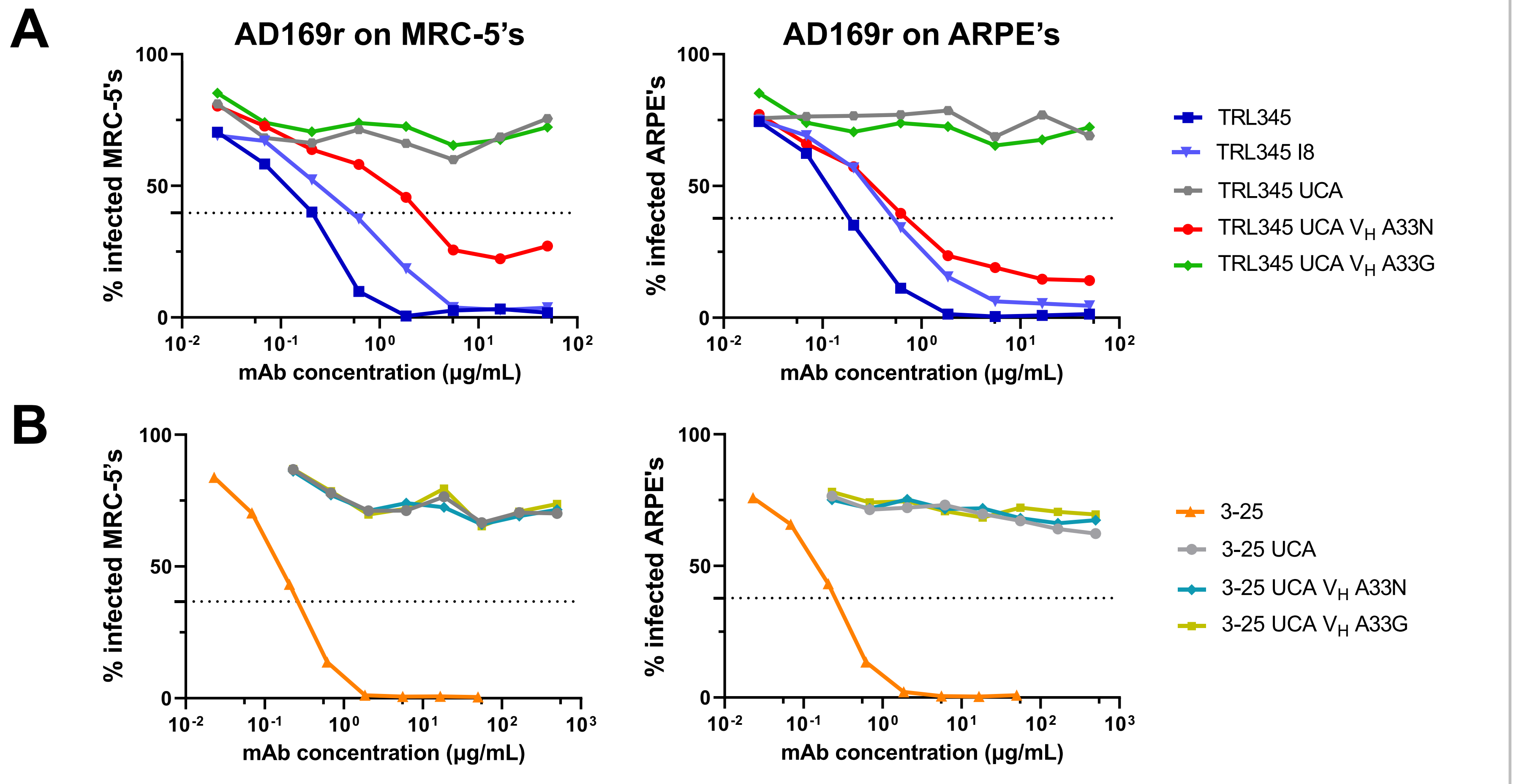

Fig. S3. Introduction of the V_H A33G mutation to the UCA of either the TRL345 or 3-25 lineages did not confer neutralizing function. We measured neutralization of the CMV strain AD169rUL131-GFP on MRC-5 fibroblasts or ARPE epithelial cells for the following mAbs:

(A) TRL345 lineage antibodies and TRL345 UCA with either the V_H A33N or A33G mutations.

(B) 3-25 lineage antibodies and 3-25 UCA with either the V_H A33N or A33G mutations. 3-25 mutant mAbs were coincubated with virus at a 10-fold higher starting concentration than all other mAbs, but even at these higher concentrations, these mAbs were non-neutralizing.
