# Supplementary material for: A single, improbable B cell receptor mutation confers potent neutralization against cytomegalovirus
Source: PLoS Pathog. 2023 Jan 20;19(1):e1011107. doi: 10.1371/journal.ppat.1011107 (PMC9891502; doi:10.1371/journal.ppat.1011107)
Supplement: S4 Fig — (PDF) [file ppat.1011107.s004.pdf]

A Heavy chains

```
                                                    33                   52a
                                               CDR1                  CDR2
TRL345 UCA    QVQLVESGGGVVQPGRSLRLSCAASGFTFSSYAMHWVRQAPGKGLEWVAVISYDGSNKYYA    60
I8            QVQLVESGGGVVQPGRSLRLSCAASGFTFSSYNMHWVRQAPGKGLEWVAVISNDGSNKYYA    60
TRL345        QVQLVESGGGVVQPGRSLRLSCAASGFTFSDYNMHWVRQAPGKGLEWVAVISIDGTYKYSA    60
3-25 UCA      QVQLVESGGGVVQPGRSLRLSCAASGFTFSSYAMHWVRQAPGKGLEWVAVISYDGSNKYYA    60
3-25          QVQLVESGGGVVQPGRSLRLSCAASGFTFSNHGLHWVRQPPGKGLEWVAVVSKDGTNEHYA    60
                                                    CDR3
TRL345 UCA    DSVKGRFTISRDNSKNTLYLQMNSLRAEDTAVYYCARDGRSVGG--FSGILDPWGQGTLVT   110
I8            DSVKGRFTISRDNSKNTLYLQMNSLRAEDTAVYYCARDGRSVGG--FSGILDPWGQGTLVT   110
TRL345        DSVAGRFSLSRDNSKNTLYLQMNSLRPDDTAIYYCARDGRSVGG--FSGILDPWGQGTLVT   110
3-25 UCA      DSVKGRFTISRDNSKNTLYLQMNSLRAEDTAVYYCAREGYCSGGSCYSGQPDYWGQGTLVT   110
3-25          DSVRGRFTISRDNSKNTLYLLMKSLRLEDTAVYYCAREGYCGDDRCYSGQPDYWGQGILVT   110

TRL345 UCA    VSS    113
I8            VSS    113
TRL345        VSS    113
3-25 UCA      VSS    113
3-25          VSS    113
```

TRL345 I8 binding sites to gB AD-2S1

3-25 binding sites to gB AD-2S1

Early, improbable mutations

B Light chains

```
                                             30                    53
                                          CDR1                 CDR2
TRL345 UCA    EIVLTQSPATLSLSPGERATLSCRASQSVSSYLAWYQQKPGQAPRLLIYDASNRATGIPA    60
I8            EIVLTQSPATLSLSPGERATLSCRASQSVGSYLAWYQQKPGQAPRLLIYDASDRATGIPA    60
TRL345        EIVMTQSPATLSLSPGDRATLSCRASQSVGSYLAWYQQKPGQAPRLLMYDSSVRATGIPA    60
3-25 UCA      EIVLTQSPATLSLSPGERATLSCRASQSVSSYLAWYQQKPGQAPRLLIYDASNRATGIPA    60
3-25          EIVLTQFPATLSLSPGERATLSCRASQSVGRYLAWYQQKPGQAPRLLIYDSSNRATGVPA    60
                                               CDR3
TRL345 UCA    RFSGSGSGTDFTLTISSLEPEDFAVYYCQQRSNWPPLTFGGGTKVEIK    107
I8            RFSGSGSGTDFTLTISSLEPEDFAVYYCQQRSNWPPLTFGGGTKVEIK    107
TRL345        RFSGSGSGTDFTLTISSLEPEDFAVYYCQQRNNWPPLTFGGGTKVEIK    107
3-25 UCA      RFSGSGSGTDFTLTISSLEPEDFAVYYCQQRSNWPPLTFGGGTKVEIK    107
3-25          RFSGSGSGTDFTLSISSLEPEDFAVYFCQQRSHWPPLTFGGGTKVEIK    107
```

Fig. S4. Sequence alignment of anti-gB AD-2S1 mAbs from the TRL345 and 3-25 lineages. The TRL345 UCA, I8, and mature mAb were aligned with the 3-25 UCA and mature mAb for the (A) heavy chains and (B) light chains. Amino acid numbering is in the Kabat scheme. CDR regions are underlined. Blue boxes indicate the contact sites for the TRL345 I8 antibody to gB AD-2S1 peptide by crystal structure, and the yellow boxes indicate the contact sites for the 3-25 mature antibody to gB AD-2S1. Blue letters indicate amino acids that differ between the TRL345 UCA and 3-25 UCA. Red letters indicate amino acid mutations from the respective UCAs of each lineage.
