# Supplementary material for: A single, improbable B cell receptor mutation confers potent neutralization against cytomegalovirus
Source: PLoS Pathog. 2023 Jan 20;19(1):e1011107. doi: 10.1371/journal.ppat.1011107 (PMC9891502; doi:10.1371/journal.ppat.1011107)
Supplement: S5 Fig — (PDF) [file ppat.1011107.s005.pdf]

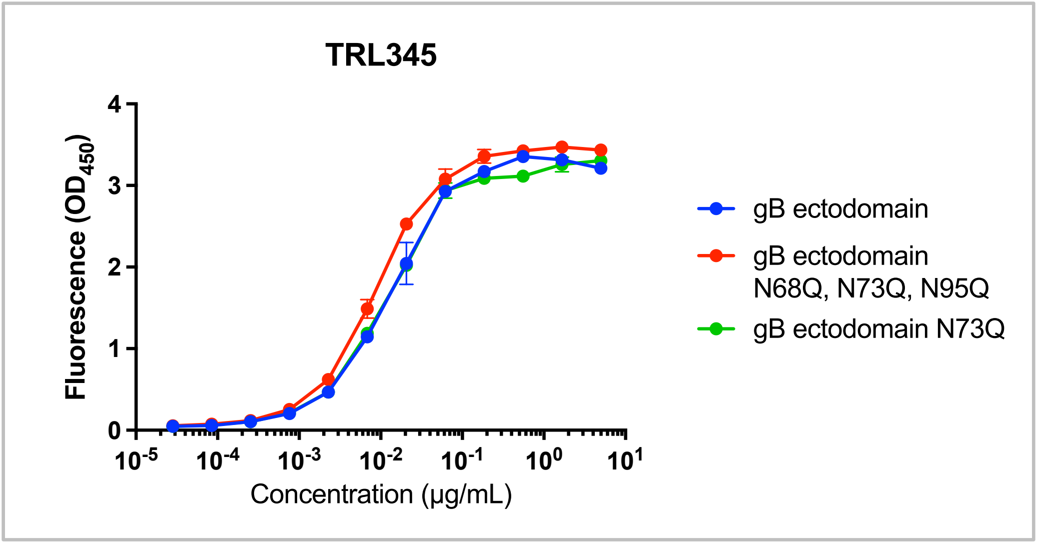

Fig. S5. Binding of TRL345 to gB ectodomain with and without mutations at N-glycosylation sites. CMV gB ectodomain protein was produced with and without mutations at known N-glycosylation sites N68, N73, and N95, of which N68 and N73 are in the AD-2S1 region. Binding of TRL345 (5 µg/mL) to these gB ectodomain proteins was measured by ELISA. Mutation of the gB glycan sites, including the gB N73 residue which is a contact site for TRL345 and 3-25, did not significantly affect TRL345 binding.
