# Supplementary material for: A single, improbable B cell receptor mutation confers potent neutralization against cytomegalovirus
Source: PLoS Pathog. 2023 Jan 20;19(1):e1011107. doi: 10.1371/journal.ppat.1011107 (PMC9891502; doi:10.1371/journal.ppat.1011107)
Supplement: S1 Table — (PDF) [file ppat.1011107.s006.pdf]

Table S1. Compiled binding and neutralization responses for TRL345 lineage mAbs and 3-25 mature mAb.

Assay	***ELISA***		***SPR***								***Cell-associated gB binding***	***Whole virion binding***	***Neutralization on fibroblasts***				***Neutralization on epithelial cells***
	gB AD-2	*gB ectodomain*	*gB AD-2*				*gB ectodomain*				*Area under the curve (AUC) for % gB-transfected cell binding*	*AUC for TB40/E whole virion binding*	*Towne IC_{50} (μg/mL)*	*AD169rUL131-GFP IC_{50} (μg/mL)*	*Toledo IC_{50} (μg/mL)*	*Average IC_{50} (μg/mL)*	*AD169rUL131-GFP IC_{50} (μg/mL)*
mAb	*EC_{50} (ng/mL)*	*EC_{50} (ng/mL)*	*k_a (10^6/ M*s)*	*k_d (1/ks)*	*K_d (nM)*	*Chi² (RU²)*	*k_a (10^6/ M*s)*	*k_d (1/ks)*	*K_d (nM)*	*Chi² (RU²)*							
TRL345	*3.3*	*4.4*	*151.6*	*5.88*	*0.29*	*0.61*	*12.90*	*0.025*	*0.81*	*0.59*	*151.6*	*3.20*	*0.21*	*0.24*	*0.19*	*0.21*	*0.3*
MAB343	*8.0*	*9.6*	*161.1*	*0.64*	*1.57*	*1.02*	*16.00*	*0.013*	*1.92*	*1.29*	*161.1*		*0.35*	*0.23*	*0.22*	*0.27*	*0.23*
I1	*2.6*	*4.0*	*142.7*	*4.16*	*1.59*	*1.46*	*14.10*	*0.013*	*0.92*	*0.72*	*142.7*	*3.04*	*0.4*	*0.31*	*0.25*	*0.32*	*0.4*
MAB309 (I2)	*3.3*	*5.5*	*124.3*	*7.84*	*3.77*	*2.10*	*12.60*	*<0.01*	*0.79*	*0.83*	*124.3*		*0.79*	*0.73*	*0.64*	*0.72*	*1.02*
MAB318	*2.2*	*3.6*	*132.6*	*6.44*	*3.68*	*1.24*	*11.90*	*<0.01*	*0.84*	*0.83*	*132.6*		*0.63*	*0.58*	*0.49*	*0.57*	*0.63*
MAB310 (I3)	*2.3*	*3.8*	*187.1*	*19.80*	*3.11*	*2.43*	*13.50*	*<0.01*	*0.74*	*0.67*	*187.1*		*0.31*	*0.23*	*0.22*	*0.25*	*0.31*
I4	*2.5*	*4.6*	*129.7*	*5.68*	*1.85*	*1.49*	*15.30*	*0.011*	*0.71*	*1.20*	*129.7*	*3.37*	*0.37*	*0.3*	*0.24*	*0.31*	*0.36*
MAB338	*3.4*	*3.9*	*136.3*	*4.27*	*1.99*	*1.10*	*13.60*	*<0.01*	*0.74*	*1.01*	*136.3*		*0.36*	*0.32*	*0.32*	*0.33*	*0.36*
MAB319	*6.5*	*4.0*	*130*	*2.03*	*0.90*	*0.69*	*11.80*	*<0.01*	*0.85*	*0.95*	*130*		*0.62*	*0.64*	*0.57*	*0.61*	*0.78*
I5	*5.2*	*4.4*	*122.6*	*18.60*	*5.52*	*0.08*	*13.90*	*<0.01*	*0.72*	*1.20*	*122.6*		*0.52*	*0.46*	*0.41*	*0.46*	*0.59*
MAB313	*2.2*	*3.5*	*154.5*	*51.20*	*4.38*	*3.35*	*13.10*	*0.011*	*0.82*	*0.88*	*154.5*		*0.26*	*0.18*	*0.18*	*0.21*	*0.2*
MAB316	*2.5*	*3.7*	*163*	*8.23*	*2.96*	*1.30*	*12.60*	*<0.01*	*0.79*	*0.93*	*163*		*0.46*	*0.42*	*0.37*	*0.42*	*0.56*
I6	*2.9*	*4.2*	*160.7*	*7.92*	*2.69*	*1.68*	*14.50*	*<0.01*	*0.69*	*1.21*	*160.7*		*0.35*	*0.31*	*0.28*	*0.31*	*0.36*
I7	*2.5*	*4.1*	*146.4*	*13.80*	*4.47*	*0.84*	*10.90*	*<0.01*	*0.92*	*1.03*	*146.4*		*0.49*	*0.39*	*0.3*	*0.39*	*0.46*
I8	*2.5*	*4.6*	*132.6*	*199.00*	*23.20*	*4.59*	*9.95*	*<0.01*	*1.01*	*1.23*	*132.6*	*2.19*	*2.78*	*0.83*	*0.83*	*1.48*	*0.79*
UCA	*377.1*	*171.7*	*97.3*	*170.00*	*27.60*	*0.04*	*8.27*	*0.264*	*31.90*	*0.74*	*97.3*	*0.28*	***50***	***50***	***50***	***50***	***50***
3-25	*3.0*	*3.7*	*148.9*	*27.00*	*4.42*	*2.28*	*12.30*	*<0.01*	*0.81*	*0.53*	*148.9*	*2.83*	*0.27*	*0.31*	*0.26*	*0.28*	*0.48*

In the neutralization assays, mAbs were run in an 8-point 3x dilution series, starting at 50 μg/mL. mAbs that did not achieve 50% inhibition of maximal infection were determined to be "non-neutralizing," and their IC_{50} values were set to 50.0 μg/mL (light gray boxes), which was the maximal concentration measured. The positive control mAb 3-25 was assessed in all assays (dark grey boxes).
