# Supplementary material for: A single, improbable B cell receptor mutation confers potent neutralization against cytomegalovirus
Source: PLoS Pathog. 2023 Jan 20;19(1):e1011107. doi: 10.1371/journal.ppat.1011107 (PMC9891502; doi:10.1371/journal.ppat.1011107)
Supplement: S2 Table — (PDF) [file ppat.1011107.s007.pdf]

Table S2. Mutational probability analysis using ARMADiLLO algorithm.

	Heavy chain			Light chain			mAb total
mAb	# nt mutations	# AA mutations	# AA mutations (Pr<0.02)	# nt mutations	# AA mutations	# AA mutations (Pr<0.02)	# AA Mutations (P<0.02)
MAB345	30	12	6	7	12	2	8
MAB343	21	13	5	4	8	3	8
I1	16	10	5	4	7	3	8
MAB309 (I2)	25	18	7	8	16	2	9
MAB318	25	18	8	9	18	2	10
MAB310 (I3)	23	15	6	7	12	2	8
I4	16	10	5	3	6	2	7
MAB338	40	19	6	6	16	4	10
MAB319	30	14	5	4	9	2	7
I5	21	10	5	3	6	2	7
MAB313	38	21	8	8	16	3	11
MAB316	35	20	6	7	14	5	11
I6	30	17	6	6	13	4	10
I7	12	5	3	2	4	2	5
I8	5	2	2	2	3	2	4
UCA	0	0	0	0	0	0	0
