# Supplementary material for: A single, improbable B cell receptor mutation confers potent neutralization against cytomegalovirus
Source: PLoS Pathog. 2023 Jan 20;19(1):e1011107. doi: 10.1371/journal.ppat.1011107 (PMC9891502; doi:10.1371/journal.ppat.1011107)
Supplement: S3 Table — (PDF) [file ppat.1011107.s008.pdf]

Table S3. X-ray crystallographic data collection and refinement statistics.

	I8 Fab + gB AD-2S1
**PDB ID**	**----**
**Data collection**	
Space group	*P 21 21 21*
Cell dimensions	
a, b, c (Å)	66.9, 74.7, 104.9
α, β, γ (°)	90.0, 90.0, 90.0
Wavelength (Å)	0.9792
Resolution (Å)	66.88-1.80 (1.84-1.80)
Unique reflections	48,200 (2,826)
R_{merge}	0.061 (0.130)
R_{pim}	0.040 (0.087)
I/σI	17.2 (9.6)
$CC_{1/2}$	0.997 (0.982)
Completeness (%)	97.5 (97.7)
Multiplicity	6.0 (6.0)
Wilson *B*-factors (Å^2)	13.4
**Refinement**	
Resolution	56.39-1.80 (1.84-1.80)
Unique reflections	48,141 (2,634)
R_{work}/R_{free} (%)	15.0/17.4 (17.9/20.7)
No. atoms	
Protein	3361
Water	723
Ligands	6
B-factors (Å^2)	
Protein	15.3
Water	31.7
Ligands	14.0
R.m.s. deviations	
Bond lengths (Å)	0.01
Bond angles (°)	0.77
Ramachandran	
Favored (%)	98.6
Allowed (%)	1.4
Outliers (%)	0.0
