# Supplementary material for: A single, improbable B cell receptor mutation confers potent neutralization against cytomegalovirus
Source: PLoS Pathog. 2023 Jan 20;19(1):e1011107. doi: 10.1371/journal.ppat.1011107 (PMC9891502; doi:10.1371/journal.ppat.1011107)
Supplement: Attachment — Submitted filename: CMV gB AD2_Response to reviewers_revision.docx [file ppat.1011107.s009.docx]

Thank you for the opportunity to revise and resubmit our manuscript “A single, improbable B cell receptor mutation confers potent neutralization against cytomegalovirus” for consideration of publication. We greatly appreciated the feedback from the three reviewers that has helped us to improve our manuscript.

**Reviewer's Responses to Questions**

**Part I - Summary**
Please use this section to discuss strengths/weaknesses of study, novelty/significance, general execution and scholarship.

Reviewer #1: In this study, Jenks et al. investigated the genealogy of B cells encoding potently neutralizing anti-gB AD-2S1 antibodies from a single patient. By computational analysis, they inferred an unmutated common ancestor (UCA). Along this prediction, they characterized the binding and function of early lineage ancestors and found that a single amino acid V(H) mutation A33N conferred broad neutralizing activity to the non-neutralizing UCA antibody. The A33N was predicted to be an improbable mutation rarely generated by somatic hypermutation. Crystallographic studies indicated that this mutation improved critical contacts with the gB AD-2S1 epitope.

Overall, the study is well designed and clearly presented. The data are convincing and generally support the conclusions (one important exception is mentioned below). The study provides a plausible explanation for the fact that neutralizing antibodies directed against AD-2S1 are found rather infrequently in HCMV-infected individuals and not at all in individuals vaccinated with a gB vaccine. This information might indeed be important for rational vaccine design. One important limitation of the study is the fact that the antibody genealogy was analyzed in a single patient. Hence it is impossible to decide whether the conclusions drawn from this patient’s antibodies are widely applicable or specific for a subset of the human population. The authors state repeatedly that their study demonstrates the feasibility lineage-based vaccine design to target induction of CMV gB AD-2S1-specific potently neutralizing antibodies. This is an overstatement. They did not show that B cell lineage selection in a vaccinated individual can actually be improved using the data from the present study.

We appreciate this detailed summary and feedback. We have removed the indicated overstatements about the feasibility of B cell lineage-based vaccine design to target induction of gB AD-2S1-specific neutralizing antibodies in the Author Summary (lines 52-54 in Revised Article with Changes Highlighted doc), Introduction (lines 151-153), and Discussion (lines 378-380, 406-407, 421-424, 431).

Reviewer #2: This is an interesting study that addresses an important immunological question concerning the maturation of antibody responses against a key neutralising epitope in gB - AD2.
In general, the project is performed well and presented in a logical manner. although based on a single donor this is mitigated by the well established highly restricted VDJ usage for AD2.
In this reviewer's opinion there are not major weaknesses although there does appear to be obvious experiments missing which should temper some of the interpretations made. This can be solved with the addition of some additional simple experiments and/or more refined interpretations of the data.

We appreciate this thoughtful response. We have added additional data, including antibody binding to CMV whole virion (see full response in Part II below).

Reviewer #3: In this interesting paper by Jenks and colleagues, novel observations are reported that have potentially a high impact on the as-yet elusive goal to develop a CMV vaccine. Since CMV is a leading cause of infant hearing loss and developmental disability, there is a great interest (particularly among pediatric infectious diseases specialists) in developing a CMV vaccine. It has been presumed that neutralizing antibodies elicited by vaccination will be key in protection, although the senior (corresponding) author of this paper has been a leader in elucidating the role of non-neutralizing antibodies in protection against CMV infection, particularly in the context of subunit gB vaccination using the first-generation gB/MF59 vaccine.

These studies extend previous work about gB vaccine. Of longstanding interest has been the elucidation of the importance of antibody against key epitopes in the gB molecule, since this can inform and direct subunit vaccine design. The AD-2 epitope is of unique interest, because of the fact that anti-AD2 responses are, and because AD-2 responses (curiously, only augmented by vaccination in those who had pre-vaccine AD-2 antibody) correlate with reduction of viremia in some vaccine studies, e.g., Baraniak, doi: 10.1093/infdis/jiy102. gB-based candidate vaccines have yet to elicit robust responses against this region and a substantial proportion of patients do not make AD-2 antibody. Lantto showed years ago that residues in the first complementarity determining region of both the heavy and the light chain were involved in determining the AD-2 specificity and that key mutations in the germ-line sequence were required for effective interaction with this epitope. Human germ-line IGHV3-30 and IGKV3-11 genes were the only V genes that participated in an AD-2 specific Ab response. He proposed that the inability of the human germ-line gene-encoded Ab repertoire to directly recognize this antigenic determinant resulted in poor immunogenicity in vivo.

Against this background, Jenks mapped the genealogy of B cells encoding potently neutralizing anti-gB AD-2 antibodies and found that a single amino acid heavy chain mutation, A33N, rarely generated by somatic hypermutation machinery, conferred broad CMV neutralization. Structural studies revealed that this mutation mediated key contacts with the gB AD- 2 epitope.

The scientific flow the paper is impeccable and elegant. Appropriate controls are included, and conclusions are supported by the data. The best, most cutting-edge techniques are used. The methodology and, in general, the interpretations are beyond reproach.

I would like to raise threes for the authors to consider and expound upon. First, the implications of the work – the assertion that the solving of the atomic structure of a gB AD peptide bound by an early B cell precursor mAb may represent the structure of the gB AD linear region capable of eliciting potently neutralizing antibodies in the host – maybe be over-stated. This conclusion may be prematurely speculative. Thus, this reviewer would suggest toning down the conclusions and minimizing the speculation that stabilization of the gB AD peptide in this conformation may be the next key step in the design of a next-generation gB candidate vaccine. Maybe this is true; but the authors should be careful to acknowledge potential pitfalls and uncertainties, maybe even going so far as to add a few sentences to indicate what these pitfalls and challenges may engender.

We appreciate this thorough summary and critique. We have toned down our conclusions and minimized our speculation in the Discussion. Specifically, we have removed overstatements about the feasibility of B cell lineage-based vaccine design to target induction of gB AD-2S1-specific neutralizing antibodies in the Author Summary (lines 52-54), Introduction (lines 151-153), and Discussion (lines 378-380, 406-407, 421-424, 431). We have also clarified that further studies are needed to determine whether the stabilized gB AD-2 peptide would be able to elicit neutralizing lineages and to assess its role as a potential next-generation vaccine in the Discussion (lines 378-380).

A second, more broad criticism this reviewer has centers around the putative novelty of the finding. The finding of the single amino acid heavy chain mutation, A33N, is very exciting, but in a sense, isn’t it derivative of what Lantto already described? Previous work showed that induction of the VH A33 residue, in this case a A->D mutation, was critical for high-affinity binding. Putting aside the issue of the patentability of the discovery (not relevant to suitability of the manuscript, but still a bit of a curiosity), what makes this new discovery fundamentally distinct from the Lannto paper? This review believes the authors should comment further on this point. In this context, it’s a bit surprising that the control for the A33N mutation was A33G, and that the A33D was not generated and tested (did the authors perform the experiments described in Figure 4 with A33D? That would have been interesting).

Thank you for the opportunity to clarify the novelty of our study. The Lantto study of the gB AD-2S1 mAb ITC88 utilized CDR shuffling to determine that induction of the V_H_ A33D mutation was critical for high-affinity binding in the IGHV3-30*04 and IGKV3-11 pairing, and it is remarkable that our computational variable region evolution assessment approach identified the same mutation [1]. Importantly, their study did not assess antibody function. Consistent with these results, we determined that the V_H_ A33N mutation in the TRL345 lineage not only significantly improved binding to gB AD-2S1 peptide and gB ectodomain for the TRL345 UCA but that it also improved antigen binding for the UCA of a second donor (**Fig. 1, 2, 4**). Our study found that this mutation was sufficient to confer potent and broad neutralizing function to the non-neutralizing TRL345 UCA (**Fig. 3**). In our study, we also used crystallography to determine the structure of a gB AD-2 peptide bound to an early progenitor gB AD-2 mAb with potent neutralizing function and compared this structure with that of mature potently neutralizing gB AD-2 mAbs (**Fig. 5**).

As compared with the CDR shuffling technique used in the Lantto paper, the methods used in our paper to identify mutations associated with antibody function may be more easily adopted in future studies aimed at defining critical antibody evolution pathway towards mAbs of critical antiviral functions. We used phylogenetic inference to characterize a single lineage of gB AD-2 mAbs and identified improbable mutations that were unlikely to occur in the absence of selection. We then strategically tested *in vitro* only these early, improbable mutations, which was a fraction of all of the total mutations possible in the heavy and light chain CDRs. In our original submission, we had referenced Lantto paper in our Introduction (lines 108-110) and compared our study to the Lantto study in the Discussion (lines 383-390). We also adjusted wording in the Discussion to clarify the novelty of our study (lines 385-387).

Regarding testing of V_H_ A33G vs. V_H_ A33D, V_H_ A33G was tested because this mutation was present in multiple mature gB AD-2 mAbs, including 3-25, which was studied in this paper.

The final point that this reviewer would raise has to do with vaccine design. It is straightforward conceptually (although perhaps not at all “easy”) to envisage how these findings might usher in a re-design of therapeutic monoclonal antibodies. In this context, a brief reference to the failed studies of CMV Ig, both polyclonal pooled sera (cytogam, cytotech) and monoclonals, is worth considering. For example, would the Hughes study (DOI: 10.1056/NEJMoa1913569; please consider citing this paper and commenting!) finally “work this time” with this newly reconfigured moab? But, more importantly, HOW, precisely, would the authors envisage this being generated into an ACTIVE vaccine for gB (as opposed to “passive” vaccination with a moab)? After all, we are born with the VDJ recombinatorial machinery we are born with. How could an immunization be designed that would instruct the vaccinated host on how to generate the proper heavy chain IgG sequence? If the authors are really thinking this exciting new data will inform how we design and administer a vaccine, please tell us how the responses of the recipient’s heavy chain sequences can be so modified.

Thank you for bringing up this important paper. The Hughes study found that administration of CMV hyperimmune globulin to pregnant women diagnosed with primary CMV infection, administered after seroconversion, did not reduce the incidence of a composite of congenital CMV infection or perinatal death compared to placebo [2]. CMV hyperimmune globulin is an IVIG product pooled from CMV-seropositive donors, and the product is known to have *in vitro* neutralizing function [3]. In preparation of this product, donors were not screened specifically for antibodies to CMV gB AD-2S1, and previous studies have shown that only ~50% of naturally CMV-infected individuals have detectable circulating anti-gB AD-2S1 antibodies [4]. Given that gB AD-2S1 antibodies have been associated with protection from CMV vertical transmission [5], future studies should assess whether a CMV IVIG product from individuals with known gB AD-2S1 antibodies or additional administration of gB AD-2S1 mAbs could improve this product and potentially confer protection.

Regarding vaccine development, we anticipate that the atomic structure of the gB AD-2S1 peptide bound to an early progenitor B cell may represent an antibody-antigen engagement capable of initiating neutralizing lineages (Discussion lines 375-380). Future studies could assess whether this immunogen designed to elicit high affinity UCA engagement and critical mAb intermediate binding can induce specific, favorable heavy chain mutations. We also recognize that there may be low inherent frequency of these B cell precursors across donors, which could be better evaluated in future unbiased B cell repertoire sequencing studies (lines 424-425). The frequencies of these B cell precursors cannot be changed, but favorable, functional mutations may be able to be elicited with a vaccine antigen dose and frequency effect.

**Part II – Major Issues: Key Experiments Required for Acceptance**
Please use this section to detail the key new experiments or modifications of existing experiments that should be absolutely required to validate study conclusions.

Generally, there should be no more than 3 such required experiments or major modifications for a "Major Revision" recommendation. If more than 3 experiments are necessary to validate the study conclusions, then you are encouraged to recommend "Reject".

Reviewer #1: 1. The genealogy of neutralizing AD-2S1 antibodies was studied in a single patient, and the crucial A33N mutation was predicted to be improbably. This raises the question of whether neutralizing antibodies generally require unlikely mutations in most or all individuals. A previous study found that non-germ-line encoded residues are critical for effective antibody recognition of the poorly immunogenic gB AD-2 epitope (Lantto et al., Eur J Imunol 2002, PMID 12115649). Are these mutations also predicted to be improbable?

We appreciate this comment. The Lantto 2002 paper found that in the derived UCA for the ITC88 gB AD-2 mAb, the heavy chain mutation A33D (numbered as A34D in their manuscript) in the IGHV3-30*04 allele was “critical” to antigen binding [1]. We derived the ITC88 UCA then applied the ARMADiLLO computational algorithm to simulate somatic hypermutation. We found that the V_H_ A33D mutation had a 1.05% probability of occurring in the absence of B cell selection, making it an improbable mutation. The Lantto paper also found that the light chain mutations S30G and S31G in the IGKV3-11 chain conferred moderate antigen binding. The TRL345 antibody used the same light chain as ITC88, so the probabilities of these mutations V_L_ S30G and S31G mutation were reported in our manuscript (**Fig. 1**)**.** We found that each mutation had <2% probability of occurring in the absence of selection (**Fig. 1**).

We also estimated the probability of the V_H_ A33G mutation occurring in the lineage development of the 3-25 mAb, derived from the IGHV3-30-3*01 allele. We found that V_H_ A33G was also improbable, occurring with an estimated frequency of just 1.94%. In combination with the frequency estimates for the mutation VH A33N in the TRL345 lineage, our data indicated that across multiple donors, functional mutations at the heavy chain residue 33 were statistically improbable and likely occurred under pressure of selection.

We have edited the manuscript to further highlight this information in the text (lines 274-282).

Reviewer #2: 1. in this study the authors have measured antibody binding to multiple different gB targets but seem to have missed the 2 most physiological.
Firstly, they have not assessed binding to virion (unless i have missed it) which is the main target of the antibody. Of course they measure functional activity by neutralisation but the data presented suggests the higher affinity of the antibody for AD2 is the explanation. I suspect it is true but then the antibodies should bind virions with higher affinity. It is possible they don't and there is another functional explanation - it is worth confirming this.
Secondly, they look at AD2 binding in the context of plasma membrane - with actually a very modest effect. Thus AD2 in membranes may be presented differently (including virion membranes) and so is relevant to the point above. Secondly, they have never looked at gB expressed by HCMV (i.e. in infected cells) - it is transfected gB. There is likely a lot of differences in an infected cell that could change gB on the surface compared to transfected gB. I do not suggest it as a replacement for transfection but as an addition.

We appreciate this insightful feedback and agree that gB as expressed on the surface of a CMV virion may differ from that as expressed independently. We have added an additional experiment to measure antibody binding to CMV whole virions by ELISA (**Fig. 2E,** lines 191-193). Consistent with the mAb binding patterns to gB AD-2 and whole gB, our results showed that the UCA had limited binding to whole CMV virions but high binding was acquired in the transition from the UCA to I8 and further refined along lineage maturation (lines 191-193).

Although we did not directly measure binding to CMV infected cells, in this paper, in sum, we measured antibody binding to many forms of the epitope, including gB AD2-S1 peptide, full-length gB protein, gB expressed on the surface of a cell, and gB on whole CMV virions, and antibody neutralization function against multiple strains on two cell types.

2. Epithelial cell neutralisation data - I think using the AD169 repaired UL131 virus really is not the virus to use here. A clinical isolate with epithelial cell tropism should be used. Again, we are learning that genes in ULb' could modulate virion content etc and this again could affect the biology of gB. It seems strange given all we have learnt about CMV genetics versions of AD169 virus are still used beyond HFFs.

We appreciate this insight. Unfortunately, wild-type CMV or clinical strains are not readily propagated in vitro to produce large stocks for neutralization assays, so it is the standard in the field to use lab strains derived from clinical strains. We intentionally used the AD169 repaired UL131 virus, as repair of the mutated UL131 gene restored the ability of this virus infect both epithelial and endothelial cells [6], allowing for comparisons of mAb function between the same virus in multiple cell types. This virus strain was used as a prototype virus for this study to determine the minimal mutations required for neutralizing function, as the breadth of TRL345 has been established previously [7].

Reviewer #3: The only experiment I would consider-although this reviewer does not view it as essential-would be to repeat the assays described in Figure 4, but with an A33D mutation in addition to the A33G control.

We agree this suggestion would be interesting. However, we chose not to produce the TRL345 UCA with this mutation as it was not a naturally occurring mutation in the lineage and is likely a distinct pathway for mAb evolution to engage the tyrosine.

**Part III – Minor Issues: Editorial and Data Presentation Modifications**
Please use this section for editorial suggestions as well as relatively minor modifications of existing data that would enhance clarity.

Reviewer #1: 2. The authors talk a lot about B cell lineage-targeted vaccine design without clearly explaining how it works. The concept should be explained to the reader in sufficient detail. It is important for the reader to understand that the mutation frequency within B cells cannot be changed (i.e., an unlikely mutation will remain unlikely), but tweaking the selection of suitable B cell lineages might be possible.

Thank you for this feedback. We have further clarified our description of B cell lineage-based vaccine design in lines 115-125. We have also further explained that the mutational frequency is fixed but that mutations may be able to be selected for by binding to high-affinity immunogens (lines 132-134).

3. HCMV gB has four neutralizing antigenic domains. If it is so difficult to elicit neutralizing antibodies against AD-2S1, wouldn’t it be better to focus on one of the other domains rather than using the B cell lineage-directed approach proposed here?

This reviewer raises a great point. Although there are four neutralizing domains in gB, multiple immune correlate studies have implicated antibodies specifically against gB AD-2S1 region in protection from CMV disease in multiple clinical contexts (described in the Introduction lines 76-85). We focused on the elicitation of these potently neutralizing antibodies in our study specifically because they have been challenging to elicit with previous vaccine candidates and poorly elicited in natural infection [4]. The function of the gB AD-2S1 region in the full length protein is not yet fully understood, but a recently published crystallographic structure of the prefusion gB suggested that the CMV gB AD-2S1 region was responsible for binding to gH/gL in the pentameric complex and that this interaction may have a role in triggering gB-mediated cell entry [8, 9]. Notably, in this prefusion structure, the gB AD-2S1 region could not be resolved, but when the structure of gH/gL in the pentameric complex was modeled in contact with prefusion gB, it was compatible with gH binding gB in the glycan-free hollow [8].

We have added some of this additional background information about the potential role of gB AD-2S1 in gH/gL binding and cell entry to the Introduction (lines 87-90), in addition to the reference in the Discussion (lines 374-376).

4. Parts of the discussion are somewhat repetitive. The term “established the feasibility of B cell lineage-based vaccine design” is used very often throughout the manuscript, and it is an overstatement (see general comment above). This statement needs to be toned down.\

We appreciate this detailed summary and feedback. We have removed overstatements about the feasibility of B cell lineage-based vaccine design to target induction of gB AD-2S1-specific neutralizing antibodies in the Author Summary (lines 52-54), Introduction (lines 151-153), and Discussion (lines 378-380, 406-407, 421-424, 431).

Reviewer #2: 1. Identity of transfected gB - whilst gB AD2 site 1 is conserved there are still different clades of gB with nearby mutations. have the authors tried other gBs?

This is a great point – we did not try to measure binding to other gB genotypes, as gB AD-2 is highly conserved across clinical strains [10], but we did measure neutralization function against three CMV strains representing different gB genotypes (**Fig. 2F, 2G, 3C**).

2. Whilst the data clearly support A33N as a key event are the other mutations completely uneccesary. Were multiple mutations made? Whilst not required they may make A33N better? Discussion point?

We produced 14 mutant mAbs with single heavy chain mutations A33N and Y52aN or light chain mutations S30G and N53D or combinations of these mutations, then we quantified mAb binding and neutralization (**Fig. 3**, Results lines 233-265). We found that only the V_H_ A33N mutation was both necessary and sufficient for antigen binding and neutralizing function, and addition of either V_L_ S30G or V_L_ N53D to V_H_ A33N in the UCA improved neutralizing function (**Fig. 3C**). However, addition of V_H_ Y52aN to V_H_ A33N decreased mAb neutralizing function. Surprisingly, the antibody with the highest neutralizing function was a UCA containing the early lineage mutations V_H_ A33N, V_L_ S30G, and V_L_ N53D but not V_H_ Y52aN, and this designer candidate antibody had higher neutralizing function than I8 itself. We have added further description of this to the Results (lines 261-263) and Discussion (lines 380-390).

Reviewer #3: No concerns.

PLOS authors have the option to publish the peer review history of their article ([what does this mean?](https://urldefense.proofpoint.com/v2/url?u=https-3A__journals.plos.org_plospathogens_s_editorial-2Dand-2Dpeer-2Dreview-2Dprocess-23loc-2Dpeer-2Dreview-2Dhistory&d=DwMGaQ&c=lb62iw4YL4RFalcE2hQUQealT9-RXrryqt9KZX2qu2s&r=98uhNil_h0gGU7c1-aceB6qINwgqERBd7cH46e-xJH8&m=1ZofQUvw9DSNAU2WTx60PtSSpJEOl_UdeGa2OCmQhLievBNisN4q4In15Ae3n4bO&s=wGikdSspQz8L8IsWmdnLWGec1yiujOrb_MC1IZvpN0s&e=)). If published, this will include your full peer review and any attached files.


If you choose “no”, your identity will remain anonymous but your review may still be made public.


**Do you want your identity to be public for this peer review?** For information about this choice, including consent withdrawal, please see our [Privacy Policy](https://urldefense.proofpoint.com/v2/url?u=https-3A__www.plos.org_privacy-2Dpolicy&d=DwMGaQ&c=lb62iw4YL4RFalcE2hQUQealT9-RXrryqt9KZX2qu2s&r=98uhNil_h0gGU7c1-aceB6qINwgqERBd7cH46e-xJH8&m=1ZofQUvw9DSNAU2WTx60PtSSpJEOl_UdeGa2OCmQhLievBNisN4q4In15Ae3n4bO&s=vq4HnXevJXuGzBFO5jfRCxlE7rLPAA3IJGL8lcmIuMo&e=).

Reviewer #1: No

Reviewer #2: No

Reviewer #3: No

Figure Files:

While revising your submission, please upload your figure files to the Preflight Analysis and Conversion Engine (PACE) digital diagnostic tool, <https://pacev2.apexcovantage.com>. PACE helps ensure that figures meet PLOS requirements. To use PACE, you must first register as a user. Then, login and navigate to the UPLOAD tab, where you will find detailed instructions on how to use the tool. If you encounter any issues or have any questions when using PACE, please email us at [figures@plos.org](mailto:figures@plos.org).

Data Requirements:

Please note that, as a condition of publication, PLOS' data policy requires that you make available all data used to draw the conclusions outlined in your manuscript. Data must be deposited in an appropriate repository, included within the body of the manuscript, or uploaded as supporting information. This includes all numerical values that were used to generate graphs, histograms etc.. For an example see here: [http://www.plosbiology.org/article/info%3Adoi%2F10.1371%2Fjournal.pbio.1001908#s5](https://urldefense.proofpoint.com/v2/url?u=http-3A__www.plosbiology.org_article_info-253Adoi-252F10.1371-252Fjournal.pbio.1001908-23s5&d=DwMGaQ&c=lb62iw4YL4RFalcE2hQUQealT9-RXrryqt9KZX2qu2s&r=98uhNil_h0gGU7c1-aceB6qINwgqERBd7cH46e-xJH8&m=1ZofQUvw9DSNAU2WTx60PtSSpJEOl_UdeGa2OCmQhLievBNisN4q4In15Ae3n4bO&s=84UtrTjW4lT6JoTc1QaKbuaQRwRbJCmHc8_WfHwfAbU&e=).

Reproducibility:

To enhance the reproducibility of your results, we recommend that you deposit your laboratory protocols in protocols.io, where a protocol can be assigned its own identifier (DOI) such that it can be cited independently in the future. Additionally, PLOS ONE offers an option to publish peer-reviewed clinical study protocols. Read more information on sharing protocols at [https://plos.org/protocols?utm_medium=editorial-email&utm_source=authorletters&utm_campaign=protocols](https://urldefense.proofpoint.com/v2/url?u=https-3A__plos.org_protocols-3Futm-5Fmedium-3Deditorial-2Demail-26utm-5Fsource-3Dauthorletters-26utm-5Fcampaign-3Dprotocols&d=DwMGaQ&c=lb62iw4YL4RFalcE2hQUQealT9-RXrryqt9KZX2qu2s&r=98uhNil_h0gGU7c1-aceB6qINwgqERBd7cH46e-xJH8&m=1ZofQUvw9DSNAU2WTx60PtSSpJEOl_UdeGa2OCmQhLievBNisN4q4In15Ae3n4bO&s=slTJAZrWxc_aX4QmYZOKKyfigo67nphIN6puhj5jnos&e=)

References:

Please review your reference list to ensure that it is complete and correct. If you have cited papers that have been retracted, please include the rationale for doing so in the manuscript text, or remove these references and replace them with relevant current references. Any changes to the reference list should be mentioned in the rebuttal letter that accompanies your revised manuscript. If you need to cite a retracted article, indicate the article’s retracted status in the References list and also include a citation and full reference for the retraction notice.

Citations

1.	Lantto J, Lindroth Y, Ohlin M. Non-germ-line encoded residues are critical for effective antibody recognition of a poorly immunogenic neutralization epitope on glycoprotein B of human cytomegalovirus. Eur J Immunol. 2002;32(6):1659-69. Epub 2002/07/13. doi: 10.1002/1521-4141(200206)32:6<1659::AID-IMMU1659>3.0.CO;2-9. PubMed PMID: 12115649.

2.	Hughes BL, Clifton RG, Rouse DJ, Saade GR, Dinsmoor MJ, Reddy UM, et al. A Trial of Hyperimmune Globulin to Prevent Congenital Cytomegalovirus Infection. N Engl J Med. 2021;385(5):436-44. Epub 2021/07/29. doi: 10.1056/NEJMoa1913569. PubMed PMID: 34320288; PubMed Central PMCID: PMCPMC8363945.

3.	Miescher SM, Huber TM, Kuhne M, Lieby P, Snydman DR, Vensak JL, et al. In vitro evaluation of cytomegalovirus-specific hyperimmune globulins vs. standard intravenous immunoglobulins. Vox Sang. 2015;109(1):71-8. Epub 2015/03/15. doi: 10.1111/vox.12246. PubMed PMID: 25766313.

4.	Finnefrock AC, Freed DC, Tang A, Li F, He X, Wu C, et al. Preclinical evaluations of peptide-conjugate vaccines targeting the antigenic domain-2 of glycoprotein B of human cytomegalovirus. Hum Vaccin Immunother. 2016;12(8):2106-12. Epub 2016/03/18. doi: 10.1080/21645515.2016.1164376. PubMed PMID: 26986197; PubMed Central PMCID: PMCPMC4994730.

5.	Bialas KM, Westreich D, Cisneros de la Rosa E, Nelson CS, Kauvar LM, Fu TM, et al. Maternal Antibody Responses and Nonprimary Congenital Cytomegalovirus Infection of HIV-1-Exposed Infants. J Infect Dis. 2016;214(12):1916-23. Epub 2016/12/08. doi: 10.1093/infdis/jiw487. PubMed PMID: 27923951; PubMed Central PMCID: PMCPMC5142097.

6.	Wang D, Shenk T. Human cytomegalovirus UL131 open reading frame is required for epithelial cell tropism. J Virol. 2005;79(16):10330-8. Epub 2005/07/30. doi: 10.1128/JVI.79.16.10330-10338.2005. PubMed PMID: 16051825; PubMed Central PMCID: PMCPMC1182637.

7.	Kauvar LM, Liu K, Park M, DeChene N, Stephenson R, Tenorio E, et al. A high-affinity native human antibody neutralizes human cytomegalovirus infection of diverse cell types. Antimicrob Agents Chemother. 2015;59(3):1558-68. Epub 2014/12/24. doi: 10.1128/AAC.04295-14. PubMed PMID: 25534746; PubMed Central PMCID: PMCPMC4325823.

8.	Liu Y, Heim KP, Che Y, Chi X, Qiu X, Han S, et al. Prefusion structure of human cytomegalovirus glycoprotein B and structural basis for membrane fusion. Sci Adv. 2021;7(10). Epub 2021/03/07. doi: 10.1126/sciadv.abf3178. PubMed PMID: 33674318; PubMed Central PMCID: PMCPMC7935361.

9.	Patrone M, Secchi M, Bonaparte E, Milanesi G, Gallina A. Cytomegalovirus UL131-128 products promote gB conformational transition and gB-gH interaction during entry into endothelial cells. J Virol. 2007;81(20):11479-88. Epub 2007/08/10. doi: 10.1128/JVI.00788-07. PubMed PMID: 17686875; PubMed Central PMCID: PMCPMC2045554.

10.	Burke HG, Heldwein EE. Crystal Structure of the Human Cytomegalovirus Glycoprotein B. PLoS Pathog. 2015;11(10):e1005227. Epub 2015/10/21. doi: 10.1371/journal.ppat.1005227. PubMed PMID: 26484870; PubMed Central PMCID: PMCPMC4617298.
